# Preventing Breast Cancer in High-Risk Women: Is There Still a Role for Oophorectomy?

**DOI:** 10.1093/jncics/pkz076

**Published:** 2019-10-04

**Authors:** Barbara M Norquist, Elizabeth M Swisher, Rachel L Yung

**Affiliations:** 1 Division of Gynecologic Oncology, Department of Obstetrics and Gynecology, University of Washington, Seattle, WA; 2 Division of Medical Oncology, Department of Medicine, University of Washington, Seattle, WA

Women at increased risk of breast and ovarian cancer have multiple reasons to consider undergoing risk-reducing salpingo-oophorectomy (RRSO). Beyond the well-established benefits of dramatically reducing the risk of developing ovarian, peritoneal, and fallopian tube cancer (collectively OC) and reducing all-cause mortality (1,2), premenopausal women are commonly counseled that removing the ovaries will also reduce their risk of breast cancer, presumably because of hormonal changes secondary to surgical menopause. Early studies estimated that RRSO provided approximately a 50% reduction in the risk of breast cancer in women with *BRCA1* and *BRCA2* mutations, particularly if performed before age 45 years (3,4). These data came into question in 2015 when a study from Heemskerk-Gerritsen et al. (5) reexamined the analytical methods these cohort studies employed to calculate the risk of breast cancer with and without RRSO and proposed steps to reduce biases. The principal change is to treat RRSO as a time-dependent variable where the time before RRSO is counted as unexposed, and the time after RRSO as exposed. Treating RRSO as a time-dependent variable addresses what is known as “immortal time bias” (6) by taking the time leading up to RRSO (during which, by condition of the study, a woman is “immortal” from breast cancer) and counting it toward the nonsurgical (unexposed) group, thus lowering the breast cancer risk estimate in the nonsurgical group. Subsequent large-cohort studies that have used these methods have demonstrated either no statistically significant reduction in breast cancer risk with RRSO (7) or a reduced risk in premenopausal *BRCA2* mutation carriers only (8).

In this issue of the Journal, Dr. Mai and colleagues (9) report on the subsequent risk of breast cancer following RRSO within the Gynecologic Oncology Group protocol 0199 (GOG-0199). GOG-0199 was a large, prospective, multi-institution study of women with either pathogenic mutations in *BRCA1* (n = 519) or *BRCA2* (n = 383), or a strong family history of breast and/or ovarian cancer (n = 1473). Women aged 30 years or older chose either immediate RRSO (n = 925) or ovarian cancer screening (n = 1453) at enrollment in a nonrandomly assigned fashion. Participants were followed for cancer outcomes for 5 years and, as just described, RRSO was treated as a time-dependent variable. Of note, women with personal history of OC were excluded; however, women with a personal history of breast cancer were not and indeed made up a large portion of the cohort (n = 1004, 42%, 31% in *BRCA* carriers and 51% in those with family history only). The primary finding was that RRSO in this cohort was not associated with a reduction in breast cancer risk, with a hazard ratio (HR) of 1.04 (95% CI = 0.64 to 1.68). Among the group of women with *BRCA1* and *BRCA2* mutations, there was a statistically nonsignificant decrease in the risk of breast cancer with a hazard ratio of 0.86 (95% CI = 0.45 to 1.67), without any statistically significant differences noted when stratified by *BRCA1* vs *BRCA2*, menopausal status, or a previous history of breast cancer.

Although these results are important, there are several features of this study that limit the ability to make strong conclusions about RRSO and breast cancer risk. The total number of incident breast cancers in this cohort was only 88 (52 within mutation carriers) limiting power to detect a difference particularly within subgroups, and many of the mutation carriers were postmenopausal (206 of 902, 22.8%), a group not expected to derive additional breast cancer risk reduction from RRSO. Prior analyses have suggested that *BRCA2* mutation carriers, with their predominance of hormone-receptor positive cancers, may derive more benefit from premenopausal RRSO (8); however, this study was underpowered to adequately assess these subgroups. Also of concern is the inclusion of women with a personal history of breast cancer when development of breast cancer was the primary outcome. One potential advantage of this approach is the ability to comment on the effect of RRSO in this population. Women with a prior history of breast cancer are typically excluded from such breast cancer prevention trials because they are fundamentally different from women without a history of breast cancer in several ways: 1) the risk of developing a secondary breast cancer is different from a primary; 2) treatments for breast cancer (such as a unilateral mastectomy, use of aromatase inhibitors) modify risk of a secondary breast cancer; and 3) they have clinically significant competing risk for mortality or recurrence of their initial cancer. Notably, ovarian suppression (medical or surgical) is a historical and more recent therapy for premenopausal breast cancer (10,11), calling into question how to understand the term *RRSO* in women with a personal history of breast cancer, because this may be both a risk reduction for OC and a treatment for breast cancer patients. Inclusion of women with a history of breast cancer may therefore bias toward the null and may explain the negative findings in this study.

Despite these limitations, this study adds to the current body of literature regarding subsequent risks of breast cancer after RRSO, suggesting that the effects of RRSO on breast cancer risk in high-risk women may be smaller than previously estimated. These data along with the more recent cohort studies treating RRSO as a time-dependent variable would argue against using an anticipated reduction in breast cancer risk as a primary driver for choosing oophorectomy. Women have many factors to weigh when considering RRSO to reduce their risk of OC, including the desire for childbearing and the consequences of surgical menopause. Interest in salpingectomy with delayed oophorectomy as an alternative to RRSO is growing (12,13), and these data offer reassurance that delaying oophorectomy is unlikely to substantially affect subsequent breast cancer risk. However, the safety of this strategy in terms of OC risk reduction remains unknown. Given the uncertain impact of RRSO on breast cancer risk, *BRCA* mutation carriers still primarily face the choice between bilateral risk-reducing mastectomy or increased screening for early detection to manage their breast cancer risk.

## Notes

Affiliations of authors: Division of Gynecologic Oncology, Department of Obstetrics and Gynecology (BMN, EMS) and Division of Medical Oncology, Department of Medicine (RLY), University of Washington, Seattle, WA.

The authors have no disclosures.
